# Incident cardiovascular disease by clustering of favourable risk factors in type 1 diabetes: the EURODIAB Prospective Complications Study

**DOI:** 10.1007/s00125-022-05698-2

**Published:** 2022-04-12

**Authors:** Soraya Soulimane, Beverley Balkau, Yakima D. Vogtschmidt, Monika Toeller, John H. Fuller, Sabita S. Soedamah-Muthu

**Affiliations:** 1 Center of Research on Psychological disorders and Somatic diseases (CoRPS), Department of Medical and Clinical Psychology, Tilburg University, Tilburg, the Netherlands; 2 Clinical Epidemiology, Université Paris-Saclay, UVSQ, Inserm, CESP, Villejuif, France; 3 Hugh Sinclair Unit of Human Nutrition, Department of Food and Nutritional Sciences, University of Reading, Reading, UK; 4 Institute for Food, Nutrition and Health, University of Reading, Reading, UK; 5 Heinrich Heine University Düsseldorf, Düsseldorf, Germany; 6 Department of Epidemiology and Public Health, University College London, London, UK

**Keywords:** Cardiovascular health metrics, HbA_1c_, Risk factors, Type 1 diabetes

## Abstract

**Aims:**

The aim of this prospective study was to examine CVD risk reduction in type 1 diabetes (1) for people with favourable cardiovascular health metrics and (2) by clustering of these metrics.

**Methods:**

Data from 2313 participants from the EURODIAB Prospective Complications Study were analysed. All had type 1 diabetes (51% men, mean ± SD age 32 ± 9 years). Seven cardiovascular health metrics were studied—smoking, BMI, physical activity, a diet score, total cholesterol/HDL-cholesterol ratio, combined systolic and diastolic BP and HbA_1c_—divided into favourable/less favourable categories. Cox proportional hazards models were used to calculate HRs (95% CIs) of incident CVD for each metric. Clusters were made by scoring each individual by the number of favourable metrics.

**Results:**

A total of 163 people developed incident CVD during a mean ± SD follow-up of 7.2 ± 1.3 years. Participants with more favourable HbA_1c_ levels of <57 mmol/mol (<7.4%) had a 37% significantly lower CVD risk than those with a less favourable HbA_1c_ (HR [95% CI] 0.63 [0.44, 0.91]), and participants with a more favourable BP (systolic BP <112 mmHg and diastolic BP <70 mmHg) had a 44% significantly lower CVD risk than participants in the less favourable BP group (HR [95% CI] 0.56 [0.34, 0.92]). There was a dose–response relation with a lower HR observed with greater clustering of more favourable metrics: people with four or more favourable metrics had an HR of 0.37 (95% CI 0.18, 0.76), adjusted for sex and age at diabetes diagnosis, compared with those with no favourable metrics.

**Conclusions/interpretation:**

Low HbA_1c_ and low BP were protective cardiovascular health metrics in our study of people with type 1 diabetes. Targeting all cardiovascular health metrics could be more effective in preventing CVD than targeting single metrics.

**Graphical abstract:**

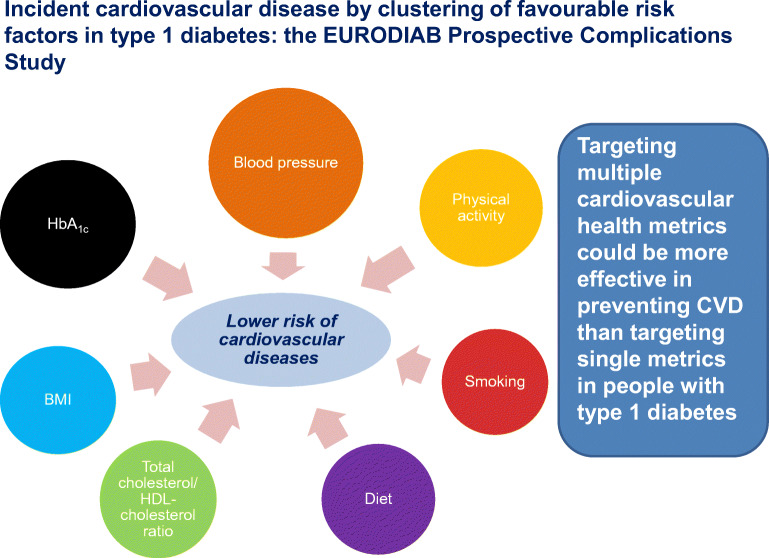

**Supplementary Information:**

The online version of this article 10.1007/s00125-022-05698-2 contains peer-reviewed but unedited supplementary material.

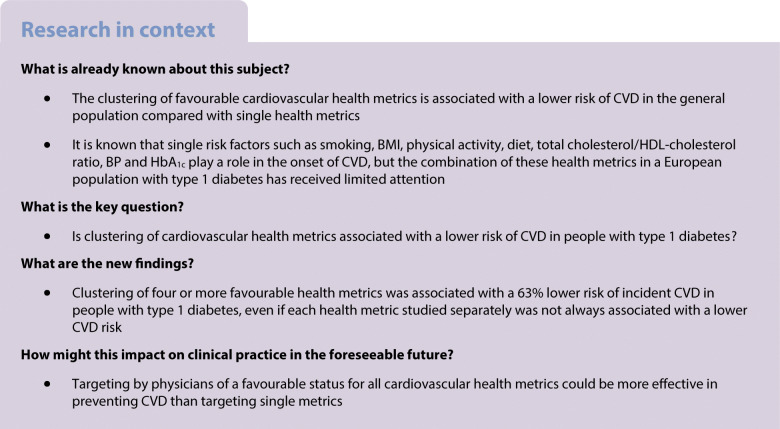



## Introduction

CVD, such as myocardial infarction and stroke, are among the most important complications in diabetes. Although there has been a decrease in the rate of cardiovascular complications in type 1 diabetes populations worldwide [1–3], these diabetic individuals have a higher risk of complications than the general population [2, 3]. Men with type 1 diabetes have a fourfold greater risk of developing cardiovascular complications than men in the general population, while women with type 1 diabetes have an up to eightfold greater risk than women in the general population [4, 5]. Nevertheless, people with type 1 diabetes with a high risk of developing cardiovascular complications may benefit from lifestyle modifications to reduce their risk of developing CVD [6].

The American Heart Association (AHA) published criteria of ideal cardiovascular health, identifying seven metrics: smoking, BMI, physical activity, diet, total cholesterol, BP and fasting plasma glucose [7]. Ideal values of each of these cardiovascular health metrics were specified, based on the literature and the recommendations of previous committees. A score is assigned based on the number of ideal cardiovascular health metrics, and studies in general populations have shown that higher scores are associated with lower risks of CVD [8–10].

Previous studies have examined ideal cardiovascular health in people with type 1 diabetes [11–16], but the criteria and the thresholds used have not always been the same as those used by the AHA. Indeed, for people with type 1 diabetes, it is more appropriate to use HbA_1c_ than fasting plasma glucose and the total cholesterol/HDL-cholesterol ratio than total cholesterol as cardiovascular health metrics [11–17].

The first studies on ideal health metrics by Alman et al [11, 12] used surrogate markers of CVD and did not focus on major cardiovascular outcomes. A cross-sectional study evaluated the prevalence of the metrics and also found that arterial stiffness (pulse wave velocity, brachial distensibility and augmentation index) decreased as the number of ideal health metrics increased [11]. A study that was both cross-sectional and prospective evaluated the prevalence and progression of coronary artery calcification (CAC) [12]. The authors found that a greater clustering of cardiovascular health metrics was associated with a lower prevalence and progression of CAC.

McCarthy et al published two cross-sectional studies on cardiovascular health metrics in people with type 1 diabetes, to explore the prevalence of the metrics and their associations with sociodemographic variables [13, 14].

Rawshani et al examined the risk of incident cardiovascular events by clustered risk factors, comparing people with and without type 1 diabetes [15]. They focused on five metrics—smoking, BP, LDL-cholesterol, HbA_1c_ and albuminuria—with risk thresholds chosen for their Swedish population. They found a graded increase in acute myocardial infarction, heart failure and stroke with an increase in the number of metrics not at target levels over a mean follow-up of 10 years [15].

In a publication from the prospective Pittsburgh Epidemiology of Diabetes Complications study, the associations between ideal cardiovascular health metrics (adapted from the AHA criteria) and incident CHD were analysed [16]. The metrics evaluated were smoking, BMI, physical activity, nutrition, total cholesterol, BP and HbA_1c_ and the follow-up period was 25 years. The study found that each additional cardiovascular health metric in the ideal range was associated with a lower risk of incident CHD (HR [95% CI] 0.68 [0.60, 0.77]) in a model adjusted for diabetes duration. The number of participants included in the analysis (*n* = 479) was limited.

The aim of this study was to evaluate the seven favourable cardiovascular health metrics and number of favourable metrics and their associations with cardiovascular events in people with type 1 diabetes from the EURODIAB Prospective Complications Study. We hypothesised that a greater clustering of these metrics would be associated with a lower cardiovascular risk, meaning that all metrics should be targeted in clinical practice [18].

## Methods

### Study design and participants

**Fig. 1:**
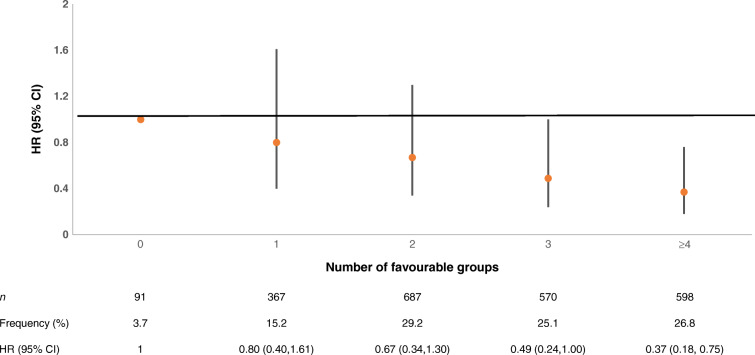
HRs (95% CIs) of cardiovascular events according to the number of the most favourable group or tertile of cardiovascular health metrics in a model on imputed data, adjusted for age at diabetes diagnosis and sex: the EURODIAB Prospective Complications Study

The EURODIAB Prospective Complications Study is a clinic-based prospective cohort study that was implemented in 31 centres in 16 European countries. Individuals with type 1 diabetes were recruited between 1989 and 1991 and sampling was stratified by age, sex and diabetes duration. Type 1 diabetes was defined as a diagnosis before 36 years, with continuous insulin therapy for at least 1 year following diagnosis [19, 20]. Participants were followed up for 7–9 years until 1999 and those without follow-up data, with prevalent CVD or without information on prevalent or incident CVD were excluded from the analysis (see electronic supplementary material [ESM] Fig. 1). This analysis includes 2313 participants.

### Outcome

The study outcome was incident CVD, which includes myocardial infarction, angina pectoris, stroke, bypass surgery and any ECG abnormalities related to possible cardiac ischaemia. Information on CVD was collected using a participant questionnaire and then checked against and completed using participants’ medical records [21].

### Cardiovascular health metrics

We based our definitions on the metrics recommended by the AHA for cardiovascular health, defining a favourable metric for each of the seven items [7]. We used HbA_1c_ rather than fasting plasma glucose, and the total cholesterol/HDL-cholesterol ratio rather than total cholesterol, as this has been shown to better predict CVD in people with type 1 diabetes than either LDL-cholesterol or non-HDL-cholesterol [17], although LDL-cholesterol was recommended for risk screening [22]. The AHA did not provide cut-off values for these variables. Data on diet and physical activity are not always collected in the same way in all studies, and previous studies have adapted their definitions [11–16]. For our European population, for continuous variables we used the favourable tertile from our study cohort to provide ideal cardiovascular risk metrics, rather than the favourable quartile, as this results in more cardiovascular events in the ideal group.

Information on smoking was obtained using a questionnaire. The favourable smoking group consisted of those who were not current smokers (never smokers and ex-smokers); this group was compared with current smokers [23].

Height and weight were measured with participants dressed in light clothing without shoes and BMI was calculated. The favourable BMI group consisted of those with values below the lower tertile (BMI <22.0 kg/m^2^).

Information on physical activity was also obtained using a questionnaire, and its intensity and frequency were categorised into four groups [19]. These values were then converted to min/week of moderate and of vigorous physical activity [19]. Participants were considered to be in the favourable physical activity group when they were in the highest tertile for moderate physical activity *or* in the highest tertile for vigorous physical activity (moderate physical activity >250 min/week *or* vigorous physical activity >60 min/week).

In order to obtain dietary data and code the food consumption of each participant, dietitians or physicians from the recruitment centres attended a 3-day workshop to determine a standardised food list from foods usually consumed in European countries, as well as portion sizes. Participants completed a 3-day dietary record (two weekdays and one Sunday). A computer program was used to determine the amount of nutrients consumed (fibre, protein, carbohydrates, total fat and saturated fat), and the total energy intake was also calculated using Atwater factors [20]. We used tertiles to determine the favourable consumption of the five nutrients: fibre ≥21.4 g/day, protein 16.0–18.8% of total energy intake, carbohydrates ≥45.7% of total energy intake, saturated fatty acids <12.1% of total energy intake and total fat ≤34.3% of total energy intake [6]. The diet score was equal to the number of favourable tertiles. Participants with three, four or five favourable dietary tertiles were compared with those with fewer than three favourable dietary tertiles.

Blood samples were taken from each participant to determine lipid and HbA_1c_ levels. Assays were carried out in a central laboratory. Lipids were assayed using standardised enzymatic methods (Boehringer Mannheim, UK) and a COBAS BIO centrifugal analyser (Roche, UK) [23]. The total cholesterol/HDL-cholesterol ratio was calculated. The favourable group consisted of those in the tertile with the lowest values (total cholesterol/HDL-cholesterol ratio <3.09).

A random zero sphygmomanometer was used to measure systolic and diastolic BP in a seated position using the right arm. Measurements were repeated after 5 min of rest and the two measurements were averaged [21]. The favourable group consisted of those in the lowest tertiles for systolic and diastolic BP (systolic BP <112 mmHg and diastolic BP <70 mmHg).

HbA_1c_ was measured using an enzyme immunoassay with a monoclonal antibody raised against HbA_1c_ (Dako, UK). These measurements were standardised and a DCCT HbA_1c_ level was calculated [23]. The group with favourable values had HbA_1c_ levels in the lower tertile (<57 mmol/mol [<7.4%]).

For the clustering of favourable cardiovascular health metrics, we attributed one point to the favourable group for each cardiovascular health metric and calculated a score for each participant relating to the number of favourable metrics. This score ranged from 0 to 7.

Concerning microvascular complications, retinopathy was diagnosed using retinal photography and the grade of retinopathy was determined at the grading centre at Hammersmith Hospital, Imperial College, London. We categorised retinopathy into two groups: no (no retinopathy) and yes (non-proliferative and proliferative retinopathy). Albuminuria was measured centrally using an immunoturbidimetric method (Sanofi Diagnostics Pasteur, USA) on a urine sample collected over 24 h. Albuminuria measured in infected urine, detected using a Nephur dipstick test for bacteria, was excluded. We categorised nephropathy into two groups: no (normoalbuminuria [≤20 μg/min]) and yes (microalbuminuria [20–200 μg/min] or macroalbuminuria [≥200 μg/min]). Peripheral neuropathy was determined by clinical examination [21].

### Statistical methods

We describe the study population and present data as mean ± SD or % (*n*), stratified by incident CVD status. Data from participants included and excluded from the study and those with and without incident CVD were compared using unpaired *t* tests or χ^2^ tests.

The percentage of missing values was zero for most variables; for age at diagnosis of diabetes and duration of diabetes it was 0.04%. Retinopathy had the highest percentage of missing data, 17.5%. Multiple imputation was used to impute missing values, to increase the statistical power and reduce bias [24]. Linear and logistic regression methods with fully conditional specification were used to impute missing values for continuous variables and categorical variables (smoking, retinopathy, albumin excretion and neuropathy). A total of 25 imputed datasets were generated. Pooled analyses were conducted on all 25 imputed datasets and the results reported in this article were retrieved from these analyses using Rubin’s methodology. Tertiles of the various cardiovascular risk factors were determined combining all of these datasets.

Analyses were conducted separately for each of the seven cardiovascular health metrics and then for the number of favourable metrics.

Univariate and multivariable Cox proportional hazards models were used to estimate the HRs of incident CVD for each favourable cardiovascular health metric. Model 1 was unadjusted; model 2 was adjusted for sex and age at diabetes diagnosis; and model 3 was used only to study each metric separately and was adjusted for sex, age at diabetes diagnosis and all other health metrics (e.g. for not current smokers, model 3 was adjusted for the metrics BMI, physical activity, diet, total cholesterol/HDL-cholesterol ratio, BP and HbA_1c_). Model 4 was performed using a complete case analysis of the original EURODIAB data (*n* = 2313) and was adjusted as in model 3. For the number of favourable cardiovascular health metrics, the model was adjusted for sex and age at diabetes diagnosis. We used a trend test by considering the number of favourable cardiovascular health metrics as a continuous variable and reporting the HR for a 1 unit increase in the number of favourable metrics. In a supplementary analysis, we also adjusted models for microvascular complications and estimated incident CVD HRs for each cardiovascular health metric and then according to the clustered health score.

Analyses were carried out using SAS software (version 9.4; SAS Institute, Cary, NC, USA).

## Results

A total of 2313 people with type 1 diabetes were included in the study. Participants had a mean ± SD age of 32 ± 9 years and a mean ± SD diabetes duration of 14 ± 9 years, and 51% of participants (*n* = 1190) were men (Table 1). Compared with those included in the study population, participants who were excluded from the analysis (*n* = 937) were older, had a longer duration of diabetes (ESM Table 1), had higher systolic and diastolic BP and had higher HbA_1c_ levels. They also consumed less fibre and carbohydrates but more saturated fatty acids and total fat. In general, the population included in the study had more healthy characteristics.

**Table 1:** Baseline characteristics of participants in the EURODIAB study of people with type 1 diabetes by incident CVD status

Variable	Total population (*n* = 2313)	With incident CVD (*n* = 163)		Without incident CVD (*n* = 2150)		*p*
			Missing		Missing	
Age (years)	32 ± 9	38 ± 11	0	32 ± 9	0	<0.0001
Men	51 (1190)	47 (76)	0	52 (1114)	0	0.22
Age at diabetes diagnosis (years)	18 ± 8	19 ± 7	0	18 ± 8	1	0.013
Diabetes duration (years)	14 ± 9	19 ± 10	0	14 ± 9	1	<0.0001
Cardiovascular health variables						
Never smokers	51 (1182)	48 (78)	1	51 (1104)	6	0.66
BMI (kg/m^2^)	23.4 ± 2.8	24.0 ± 2.8	1	23.4 ± 2.7	16	0.07
Physical activity (min/week)			0		0	
Moderate	330 ± 612	282 ± 462		330 ± 624		0.31
Vigorous	138 ± 402	87 ± 234		144 ± 408		0.07
Dietary criteria			15		190	
Fibre (g/day)	19.0 ± 7.5	18.4 ± 6.8		19.3 ± 7.5		0.16
Protein (% of energy)	17.6 ± 3.5	17.6 ± 3.5		17.6 ± 3.5		0.95
Carbohydrates (% of energy)	42.7 ± 7.1	41.7 ± 6.6		42.8 ± 7.2		0.07
Saturated fatty acids (% of energy)	13.7 ± 3.4	13.7 ± 3.1		13.8 ± 3.4		0.78
Total fat (% of energy)	37.5 ± 7.1	37.8 ± 6.5		37.5 ± 7.1		0.65
Total cholesterol/HDL-cholesterol ratio	3.8 ± 1.5	4.1 ± 1.5	1	3.8 ± 1.5	39	0.03
Blood pressure			1		7	
Systolic BP (mmHg)	120 ± 16	127 ± 19		119 ± 16		<0.0001
Diastolic BP (mmHg)	75 ± 11	76 ± 12		75 ± 11		0.39
HbA_1c_			0		16	
mmol/mol	67 ± 21	70 ± 18		67 ± 21		0.07
%	8.3 ± 1.9	8.6 ± 1.6		8.3 ± 1.9		
Other covariables						
Retinopathy	44.7 (853)	64.8 (79)	41	43.3 (774)	364	<0.0001
Nephropathy^a^	28.6 (633)	52.3 (80)	10	26.9 (553)	92	<0.0001
Neuropathy	31.5 (715)	47.8 (77)	2	30.3 (638)	43	<0.0001

Data are expressed as mean ± SD or % (*n*)

^a^Nephropathy was categorised into microalbuminuria and macroalbuminuria

Among the study population, 163 people developed incident CVD during a mean ± SD follow-up of 7.2 ± 1.3 years. As expected, compared with those without incident CVD, people with incident CVD were 6 years older, had been diagnosed with diabetes 5 years earlier, and had higher systolic BP and a higher total cholesterol/HDL-cholesterol ratio (Table 1). They also had more microvascular complications than people without CVD.

The not current smokers (never and ex-smokers) represented 68.9% of the population. The favourable physical activity group included 49.7% of participants. With regard to the dietary criteria, the favourable group included 26.1% of participants. Only 18.6% of participants were in the favourable BP group. For BMI, total cholesterol/HDL-cholesterol ratio and HbA_1c_, the favourable group was the lowest tertile group.

The HRs of cardiovascular events for the most favourable group compared with the least favourable groups for each cardiovascular health metric are presented in Table 2. In the unadjusted model (model 1), we found a statistically significant inverse association between favourable HbA_1c_ and incident CVD (HR [95% CI] 0.63 [0.44, 0.91]). This association was similar in model 2 (HR [95% CI] 0.61 [0.42, 0.87]) and remained statistically significant after further adjustment in model 3 (HR [95% CI] 0.63 [0.44, 0.91]); thus, in model 3 there was a 37% lower risk of developing incident CVD. After further adjustment for retinopathy, nephropathy or neuropathy, this strong association was at the limit of statistical significance (ESM Table 2). We also found a statistically significant inverse association between favourable BP and incident CVD in models 1 and 2 (HR [95%CI] 0.54 [0.33, 0.87]). This association remained statistically significant after adjustment for the other covariables in model 3 (HR [95% CI] 0.56 [0.34, 0.92]) and was at the limit of statistical significance after adjustment for microvascular complications.

**Table 2:** HRs (95% CIs) of incident cardiovascular events for the most favourable group or tertile vs the two less favourable groups or tertiles for each cardiovascular health metric using imputed data: the EURODIAB Prospective Complications Study

Cardiovascular health metric	Model 1	Model 2	Model 3	Model 4
Not current smoker	0.90 (0.65, 1.25)	0.92 (0.66, 1.28)	0.90 (0.64, 1.25)	0.88 (0.63, 1.22)
BMI	0.79 (0.57, 1.11)	0.79 (0.57, 1.11)	0.93 (0.65, 1.32)	0.74 (0.53, 1.03)
Physical activity	0.71 (0.52, 0.97)	0.76 (0.55, 1.04)	0.77 (0.56, 1.06)	0.77 (0.53, 1.13)
Diet	0.79 (0.54, 1.15)	0.79 (0.54, 1.15)	0.80 (0.55, 1.17)	0.92 (0.65, 1.30)
Total cholesterol/HDL-cholesterol ratio	0.80 (0.56, 1.12)	0.76 (0.53, 1.07)	0.90 (0.63, 1.29)	0.96 (0.67, 1.38)
Blood pressure	0.54 (0.33, 0.87)	0.54 (0.33, 0.87)	0.56 (0.34, 0.92)	0.55 (0.33, 0.91)
HbA_1c_	0.63 (0.44, 0.91)	0.61 (0.42, 0.87)	0.63 (0.44, 0.91)	0.65 (0.45, 0.94)

Model 1: unadjusted

Model 2: adjusted for age at diabetes diagnosis and sex

Model 3: adjusted as in model 2 plus for the other cardiovascular health metrics. For example, to estimate the HR of CVD for never smokers, model 3 was adjusted for age at diabetes diagnosis, sex, BMI, physical activity, diet, total cholesterol/HDL-cholesterol ratio, BP and HbA_1c_

Model 4: complete case analysis of original EURODIAB data (*n* = 2313), adjusted as for model 3

All cardiovascular health metrics were negatively associated with CVD incidence; however, with the exception of HbA_1c_ and BP, these associations were not statistically significant. Using the EURODIAB data on complete cases, the HRs in model 4 were similar to those in models 1–3 (Table 2).

The higher the number of favourable cardiovascular health metrics the lower the risk of incident CVD (*p*_trend_ <0.0001), with a 20%, 33%, 51% and 63% lower risk for clusters of one, two, three and four or more favourable cardiovascular health metrics, respectively, compared with the group without any favourable metrics (Fig. 1). People with four or more favourable metrics had an HR of 0.37 (95% CI 0.18, 0.75), whereas those with three had an HR of 0.49 (95% CI 0.24, 1.00), which is at the limit of statistical significance. For a 1 unit increase in the number of favourable metrics, the HR was 0.77 (95% CI 0.68, 0.88) in the model adjusted for age at diabetes diagnosis and sex. The HRs were similar after further adjustment for retinopathy, nephropathy and neuropathy (ESM Table 3).

## Discussion

This prospective study included 2313 people with type 1 diabetes and evaluated seven classic cardiovascular health metrics: smoking, BMI, physical activity, diet, total cholesterol/HDL-cholesterol ratio, BP and HbA_1c_. We found that low HbA_1c_ and low BP remain the most predictive for and protective against incident CVD. The group with HbA_1c_ <57 mmol/mol (<7.4%) had a 37% lower risk of developing incident CVD than those with a higher HbA_1c_, and the favourable BP group had a 44% significantly lower CVD risk than the less favourable group. Clustering of favourable risk factors was associated with a lower risk of incident CVD: participants with four or more favourable cardiovascular health metrics had a statistically significant 63% lower risk of developing incident CVD than the group without any favourable cardiovascular health metrics. An increase of one favourable metric was associated with a 23% lower risk of incident CVD.

The association between HbA_1c_ level and incident CVD has been demonstrated previously in type 1 diabetes [25, 26]. The DCCT/EDIC study found that, after 30 years’ follow-up, incident CVD risk decreased by 28% for a 10% decrease in mean HbA_1c_ (HR [95%CI] 0.72 [0.63, 0.81]) [25]. There was a statistically significant association between an increase of 5 mmol/mol units in HbA_1c_ and higher CVD occurrence in a Swedish study (HR [95%CI] 1.24 [1.18, 1.31] for a diabetes duration between 0 and 19 years) [26]. Our findings confirm the association between HbA_1c_ level and CVD events, with a strong and statistically significant association between the favourable HbA_1c_ metric (HbA_1c_ <57 mmol/mol [<7.4%]) and lower CVD risk (HR [95%CI] 0.63 [0.44, 0.91]) compared with higher HbA_1c_ levels. The HRs for HbA_1c_ did not change after adjustment for sex and age at diabetes diagnosis, confirming the specific association of HbA_1c_ with incident CVD.

Our results concerning the association between BP and incidence of CVD in type 1 diabetes are consistent with the results of previous studies. Indeed, in the Pittsburgh Epidemiology of Diabetes Complications Study of people with type 1 diabetes with a 25 year follow-up, the authors found that a 1 mmHg higher systolic BP was associated with a higher risk of developing incident CVD (HR [95%CI] 1.01 [1.005, 1.015]) and those with hypertension had a twofold higher risk of developing incident CVD in comparison with participants without hypertension (HR [95%CI] 2.04 [1.43, 2.91]) [27]. Shah et al also found that hypertension was associated with a twofold higher risk of developing incident CVD after a median follow-up of 5.3 years (OR [95% CI] 2.09 [1.59, 2.75]) [28]. We found that participants in the most favourable BP group had almost half the risk of developing incident CVD compared with participants in the less favourable BP group (HR [95%CI] 0.56 [0.34, 0.92]).

The results of previous studies concerning smoking and the risk of incident CVD in type 1 diabetes are inconsistent. Shah et al found no association between smoking and incident CVD in type 1 diabetes [28]. However, in another study, the authors found an association between smoking and higher risk of incident CVD (HR [95% CI] 1.75 [1.30, 2.37]) [27]. In our study, the lower risk of incident CVD in those not currently smoking was not statistically significant. The difference in results between studies could be due to the different outcomes measured. Indeed, Feodoroff et al analysed data according to the type of CVD outcome and sex and found a higher risk of stroke in men who were current smokers than in men who were never smokers (HR [95% CI] 1.99 [1.35, 2.94]) [29]. No significant association was found for women nor for the other CVD outcomes (CHD and heart failure).

We found no statistically significant association between favourable BMI or physical activity and a lower CVD risk. The results published in the literature differ between studies and may be different between men and women [30] or according to the particular physical activity characteristic evaluated. Indeed, in their study, Tikkanen-Dolenc et al [31] found different HRs depending on whether they studied physical activity intensity, frequency or duration. They found no association between a shorter leisure time physical activity (LTPA) duration and incident CVD risk. However, a lower intensity or frequency of LTPA was associated with a higher incident CVD risk (HR [95% CI] 1.91 [1.11, 3.28] and 1.94 [1.39, 2.71], respectively) [31].

The associations between diet and total cholesterol/HDL-cholesterol ratio and CVD risk were also not statistically significant. This may be because the participants in this study have followed a type 1 diabetes diet from a young age. It is difficult to compare our results with those published in the literature as the definitions of the dietary criteria used are very different between studies. Indeed, Devaraj et al used only three criteria to define the diet score: fibre, saturated fat and sodium intake [16].

The clustering of cardiovascular health metrics was associated with a lower incidence of CVD. Our findings align with the results of previous studies in people with type 1 diabetes even though they focused on only five metrics [11] or had outcomes that were different from our outcome [11, 12]. Alman et al found that, in adults with type 1 diabetes, for each additional ideal cardiovascular health metric, as defined by the AHA, the progression of CAC was lower (OR [95%CI] 0.77 [0.66, 0.90]) [12].

A recent study by Devaraj et al found that, after adjustment for diabetes duration, an increase of 1 unit in the ideal cardiovascular health metric score was associated with a 32% lower risk of CHD (*p*=0.01) [16]. Unfortunately, the HRs for the seven metrics were not reported individually. The authors used the AHA metrics to define favourable groups, except for HbA_1c_ and diet, for which they used their own definition (based on fibre, sodium and fat intakes), and for their physical activity variable (participation in sports or recreation only). Because the follow-up period in this study was longer than that in EURODIAB, and the duration of type 1 diabetes at baseline was higher, more participants would have developed CVD. We compared the prevalence of the ideal health metrics in the two populations (ESM Table 4). The most striking differences were that the EURODIAB cohort exhibited lower levels of recreational physical activity and had a healthier diet (but we were not able to take sodium intake into account) and lower HbA_1c_, but had higher BP. The HR for an additional 1 unit increase in ideal metrics using the Devaraj criteria in the EURODIAB cohort was 0.75 (95% CI 0.66, 0.85; *p*<0.001), compared with a HR of 0.77 (95% CI 0.68, 0.88; *p*<0.001) using our definitions, after adjustment for age at diabetes diagnosis and sex. Similarly, when we analysed the HRs for the individual cardiovascular health metrics using the Devaraj criteria, the HRs were very similar to those using our definitions (ESM Table 5). Thus, these HRs are remarkably robust when using the two different definitions of ideal cardiovascular health metrics.

Although there are few publications on type 1 diabetes that explore clustering, it has been demonstrated that the clustering of favourable AHA health metrics is associated with lower CVD and cardiovascular mortality in general populations [8–10]. In the National Health and Nutrition Examination Survey (NHANES III), participants with ideal cardiovascular health had a 69% lower risk of developing a silent myocardial infarction, as detected by ECG (OR [95% CI] 0.31 [0.12, 0.75]) [10]. Similarly, the Australian Health Survey found an inverse association between a higher number of ideal metrics and a lower risk of prevalent ischaemic heart disease. For each additional health metric the OR was 0.41 (95% CI 0.19, 0.88) for women; for men, the OR was not statistically significant (0.86 [95% CI 0.73, 1.02]) [8]. In their meta-analysis, Ramírez-Vélez et al found that, compared with zero to two cardiovascular health metrics, ideal cardiovascular health reduced the risk of incident cardiovascular events by 72% (HR [95% CI] 0.28 [0.23, 0.33]) [9].

### Strengths and limitations

One of the strengths of our study is that we used data from the EURODIAB Prospective Complications Study, a large cohort study conducted in 16 European countries, which ensured a good representation of the European population. The protocol was uniform across countries and the biological assays were carried out in central laboratories, making the results comparable. The EURODIAB study has a short follow-up period. Further, the baseline date of this study is 1989–1991 and the risk factor distributions and the thresholds we used may no longer be appropriate, as the treatment and care of people with type 1 diabetes has improved over the past 30 years. This should be considered in the interpretation of the HbA_1c_ results. Indeed, in the literature, HbA_1c_ is not associated with prevalent CVD after 45 years of age [32] and CVD risk decreases with a longer diabetes duration [26].

The causality of these cardiovascular health metrics with regard to incident CVD cannot be deduced from this study as it has an observational design with self-reported information and it did not consider residual confounders. In our analyses we did not adjust for socioeconomic factors even though they are predictive of CVD events [33, 34], as we did not have data for these variables. Indeed, the AHA criteria were defined for all populations independent of socioeconomic status.

## Conclusion

In conclusion, low values of HbA_1c_ and BP were protective against incident CVD and targeting a higher number of favourable cardiovascular health metrics that include lifestyle factors could significantly reduce the risk of CVD in type 1 diabetes. More research is necessary in large populations to study these relationships for different age classes and durations of diabetes to better identify the type 1 diabetic population for whom these measures should be used, and to determine an appropriate threshold for each cardiovascular health metric. Further studies should investigate which clusters of factors are important in promoting cardiovascular health.

## Supplementary information

ESM (PDF 85 kb)

## Data Availability

The EURODIAB Prospective Complications Study data are available from the corresponding author on approval of a research project.

## Abbreviations

AHA: American Heart Association
CAC: Coronary artery calcification
LTPA: Leisure time physical activity
