# Supplementary material for: Incident cardiovascular disease by clustering of favourable risk factors in type 1 diabetes: the EURODIAB Prospective Complications Study
Source: Diabetologia. 2022 Apr 12;65(7):1169–78. doi: 10.1007/s00125-022-05698-2 (PMC9174122; doi:10.1007/s00125-022-05698-2)
Supplement: Supplementary file 1 — (PDF 85 kb) [file 125_2022_5698_MOESM1_ESM.pdf]

ESM Table 1. *Characteristics of participants included and excluded from this study: the EURODIAB Prospective Complications Study of people with type 1 diabetes.*

	Included n=2313	Excluded n=937	P
Age (years)	32 ± 9	34 ± 11	<0.0001
Men	51 (1190)	51 (478)	0.8
Age at diabetes diagnosis (years)	18± 8	18± 8	0.06
Diabetes duration (years)	14 ± 9	16± 10	<0.0001
Cardiovascular health variables			
never smokers	51 (1182)	47 (428)	0.05
BMI (Kg/m²)	23.4± 2.8	23.6 ± 3.2	0.29
Physical activity (min/week)			
Moderate	330 ± 612	379± 636	0.04
Vigorous	138 ± 402	192± 480	0.004
Dietary criteria			
Fibre (g/day)	19.0 ± 7.5	17.0 ± 6.6	<0.0001
Protein (% of Energy)	17.6 ± 3.5	17.5 ± 3.4	0.39
Carbohydrates (% of Energy)	42.7 ± 7.1	41.8 ± 7.7	0.0034
Saturated fatty acids (% of Energy)	13.7 ± 3.4	14.5 ± 3.5	<0.0001
Total fat (% of Energy)	37.5 ± 7.1	38.9 ± 7.5	<0.0001
Total cholesterol/HDL-cholesterol ratio	3.8 ± 1.5	4.3 ± 2.1	<0.0001
Blood pressure			
Systolic BP (mmHg)	120 ± 16	124 ± 20	<0.0001
Diastolic BP (mmHg)	75 ± 11	77 ± 12	<0.0001
HbA1c			
mmol/mol	67 ± 21	70 ± 21	0.0003
(%)	8.3± 1.9	8.6 ± 1.9	
Other co-variables			
Retinopathy	44.6 (853)	51.0 (291)	0.0012
Nephropathy*	28.6 (633)	35.8 (302)	<0.0001
Neuropathy	31.5 (715)	47.8 (434)	<0.0001

Data are expressed as mean ± SD or % (n)

*Nephropathy regrouped micro and macro albuminuria.

ESM Table 2. *Hazard Ratios [95% CI] of incident cardiovascular events for the most favourable cardiovascular health metrics vs less favourable, adjusted for microvascular complications: the EURODIAB Prospective Complications Study.*

Favourable cardiovascular health metrics	Model 1	Model 2	Model 3
Not current smokers	0.91 [0.65-1.27]	0.95 [0.68-1.33]	0.92 [0.66-1.29]
BMI	1.01 [0.71-1.43]	0.90 [0.63-1.28]	0.96 [0.67-1.36]
Physical activity	0.80 [0.58-1.10]	0.75 [0.55-1.04]	0.79 [0.57-1.09]
Diet	0.80 [0.55-1.16]	0.81 [0.55-1.18]	0.78 [0.53-1.14]
Total cholesterol/HDL-c ratio	0.93 [0.65-1.34]	0.97 [0.67-1.39]	0.92 [0.64-1.32]
Blood pressure	0.63 [0.38-1.04]	0.65 [0.40-1.07]	0.58 [0.35-0.95]
HbA1c	0.68 [0.47-0.99]	0.71 [0.49-1.03]	0.68 [0.47-0.98]

Model 1 adjusted for age atf diabetes diagnosis, sex, the other cardiovascular health metrics, and retinopathy
Model 2 adjusted for age at diabetes diagnosis, sex, the other cardiovascular health metrics, and nephropathy
Model 3 adjusted for age at diabetes diagnosis, sex, the other cardiovascular health metrics, and neuropathy

ESM Table 3. *Hazard Ratios [95% CI] of incident cardiovascular events by number of favourable cardiovascular health metrics, adjusted for microvascular complications: the EURODIAB Prospective Complications Study.*

Number of favourable cardiovascular health metrics	Model 1	Model 2	Model 3	Model 4	Model 5
Zero	ref	ref	ref	ref	ref
One	0.84 [0.42-1.70]	0.80 [0.41-1.61]	0.89 [0.44-1.80]	0.76 [0.37-1.54]	0.81 [0.40-1.64]
Two	0.70 [0.36-1.35]	0.67[0.34-1.30]	0.76 [0.39-1.49]	0.69 [0.35-1.34]	0.72 [0.37-1.39]
Three	0.51 [0.25-1.02]	0.49[0.24-1.00]	0.60 [0.29-1.21]	0.55 [0.27-1.12]	0.56 [0.27-1.13]
Four or more	0.38 [0.19-0.78]	0.37 [0.18-0.76]	0.50 [0.24-1.03]	0.44[0.21-0.91]	0.43 [0.21-0.89]

Model 1 Unadjusted
Model 2 adjusted for age at diabetes diagnosis and sex
Model 3 adjusted for age at diabetes diagnosis, sex and retinopathy
Model 4 adjusted for age at diabetes diagnosis, sex and nephropathy
Model 5 adjusted for age at diabetes diagnosis, sex and neuropathy

ESM Table 4. *Prevalence of the favourable cardiovascular metrics in EURODIAB; prevalence of the ideal cardiovascular health metrics in the prospective Pittsburgh Epidemiology of Diabetes Complications (EDC) study as defined in the Devaraj et al publication and the prevalence of ideal cardiovascular health metrics in EURODIAB as defined in the Devaraj et al publication.*

Cardiovascular health metrics	EURODIAB		EDC study		EURODIAB using Devaraj et al. publication definitions	
Smoking	Not current smokers	68.9%	Never smokers	58.2%	Never smokers	51.2%
BMI	<22.0 Kg/m^2	33.3%	<25.0 Kg/m^2	64.6%	<25.0 Kg/m^2	73.1%
Physical activity	Moderate > 250 min/week<br>or vigorous > 60 min/week	49.7%	moderate+≥150 min per week<br>sport and leisure activity	39.4%	vigorous≥150 min	24.5%
Healthy diet	3-4-5 favourable diet tertiles	26.1%	3 components	1.2%	2 components*	2.0%
Normal lipids	(Total cholesterol / HDL-cholesterol) ratio < 3.09	33.3%	Total cholesterol<5.18mmol/l	64.4%	Total cholesterol<5.18mmol/l	48.9%
Low SBP/DBP	SBP < 112 mmHg and DBP < 70 mmHg	18.6%	SBP<120 mmHg and DBP < 80 mmHg	64.4%	SBP<120 mmHg and DBP < 80 mmHg	45.8%
Normal HbA1c	< 57 mmol/mol (< 7.4 %)	34.1%	<53 mmol/mol (<7.0%)	7.4%	<53 mmol/mol (<7.0%)	24.5%

**Fibre: women: fibre > 25g/day, men: fibre >38g/day*
Saturated fat < 10% Energy
We did not use sodium intake criteria

ESM Table 5. Hazard Ratios [95% CI] of incident cardiovascular events for the ideal cardiovascular health metrics as defined by Devaraj et al. on imputed data: the EURODIAB Prospective Complications Study.

Cardiovascular health metrics	Model 1	Model 2	Model 3	Model 4
Never smokers	0.87 [0.64, 1.19]	0.91 [0.66, 1.24]	0.96 [0.70, 1.31]	0.93 [0.68, 1.29]
BMI	0.71 [0.51, 0.98]	0.73 [0.52, 1.02]	0.86 [0.61, 1.20]	0.86 [0.61, 1.21]
Physical activity	0.64 [0.43, 0.96]	0.69 [0.46, 1.05]	0.78 [0.49, 1.22]	0.68 [0.45, 1.04]
Diet	0.72 [0.18, 2.92]	0.67 [0.16, 2.73]	0.62 [1.15, 2.54]	0.60 [0.14, 2.46]
Total cholesterol	0.76 [0.56, 1.04]	0.81 [0.59, 1.11]	1.03 [0.74, 1.44]	1.06 [0.76, 1.47]
Blood pressure	0.48 [0.34, 0.67]	0.47 [0.33, 0.65]	0.49 [0.34, 0.69]	0.50 [0.35, 0.71]
HbA1c	0.53 [0.34, 0.83]	0.51 [0.33, 0.79]	0.54 [0.34, 0.84]	0.53 [0.34, 0.83]

Model 1Unadjusted model
Model 2: adjusted for age at diabetes diagnosis and sex
Model 3: Model 2 + the other cardiovascular health metrics. For example, to estimate the Hazard ratio of CVD for never smokers, Model 3 was adjusted for age at diabetes diagnosis, sex, BMI, PA, diet, Total cholesterol, BP and HbA1cModel 4: original EURODIAB data, a complete case analysis n=2313, adjusted as for Model 3

ESM Fig. 1–The flowchart of the selection of participants for the study

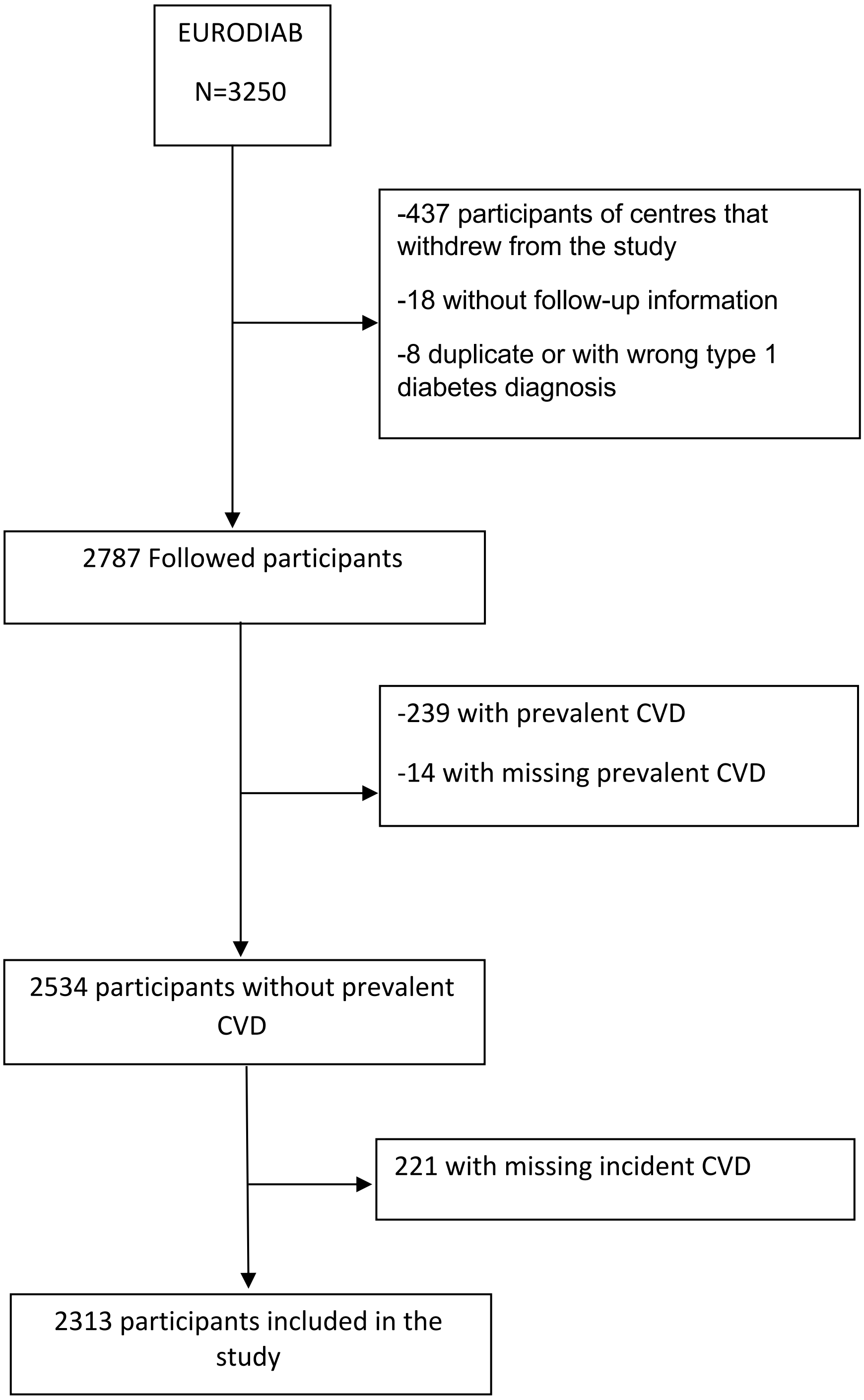

The EURODIAB Prospective Complications Study Group

B. Karamanos, A. Kofinis and K. Petrou (Hippokration Hospital, Athens, Greece); F. Giorgino, G. Picca, A. Angarano, G. De Pergola, L. Laviola and R. Giorgino (Internal Medicine, Endocrinology and Metabolic Diseases, Department of Emergency and Organ Transplantation, University of Bari, Italy); C. Ionescu-Tirgoviste, A. Coszma and C. Guja (Clinic of Diabetes, Nutrition and Metabolic Diseases, Bucharest, Romania); M. Songini, M. Pedron, S. Pintus and M. Fossarello (Diabetes Unit, Ospedale San Michele, Cagliari, Italy); A. Casu (Diabetes Service, ISMETT-IRCCS, Palermo, Italy); J. B. Ferriss, G. Grealy and D. O'Keefe (Cork University Hospital, Ireland); M. Toeller and C. Arden (Diabetes Research Institute, Heinrich Heine University, Düsseldorf, Germany); R. Rottiers, C. Tuyttens and H. Priem (Ghent University Hospital, Belgium); P. Ebeling, M. Kylliäinen and V. A. Koivisto (University Hospital of Helsinki, Finland); B. Idzior-Walus, J. Sieradzki, K. Cyganek and B. Solnica (Department of Metabolic Diseases, Jagiellonian University, Krakow, Poland); H. H. P. J. Lemkes and J. C. Lemkes-Stuffken (Leiden University Medical Centre, the Netherlands); J. Nunes-Correa, M. C. Rogado, L. Gardete-Correia, M. C. Cardoso, A. Silva, J. Boavida and M. Machado Sa Marques (Portuguese Diabetes Association, Lisbon, Portugal); G. Michel, R. Wirion and S. Cardillo (Centre Hospitalier de Luxembourg); G. Pozza, R. Mangili and V. Asnaghi (Ospedale San Raffaele, Milan, Italy); E. Standl, B. Schaffler, H. Brand and A. Harms (City Hospital Schwabing, Munich, Germany); D. Ben Soussan, O. Verier-Mine, P. Fallas and M. C. Fallas (Centre Hospitalier de Valenciennes, France); J. H. Fuller, J. Holloway, L. Asbury and D. J. Betteridge (University College London, UK); G. Cathelineau, A. Bouallouche and B. VillatteCathelineau (Hôpital Saint-Louis, Paris, France); F. Santeusanio, G. Rosi, V. D'Alessandro, C. Cagini, P. Bottini and G. P. Reboldi (Dipartimento di Medicina Interna, Perugia, Italy); R. Navalesi, G. Penno, S. Bandinelli, R. Miccoli and M. Nannipieri (Dipartimento di Endocrinologia e Metabolismo, Pisa, Italy); G. Ghirlanda, C. Saponara, P. Cotroneo, A. Manto and A. Minnella (Università Cattolica del Sacro Cuore, Rome, Italy); J. D. Ward, S. Tesfaye, S. Eaton and C. Mody (Royal Hallamshire Hospital, Sheffield, UK); M. Borra, P. CavalloPerin, S. Giunti, G. Grassi, G. F. Pagano, M. Porta, R. Sivieri, F. Vitelli and M. Veglio (Dipartimento di Medicina Interna, Università di Torino, and ASO CTO/CRF/Maria Adelaide, Turin, Italy); N. Papazoglou and G. Manes (Papageorgiou

General Hospital, Diabetes Unit, Thessaloniki, Greece); M. Muggeo, M. Iagulli and V. Cacciatori (V. Cattedra di Malattie del Metabolismo, Verona, Italy); K. Irsigler and H. Abrahamian (Hospital Vienna Lainz, Austria); S. Walford, J. Sinclair, S. Hughes, V. McLelland and J. Ward (New Cross Hospital, Wolverhampton, UK); G. Roglic, Z. Metelko and Z. R. Pepeonik (Vuk Vrhovac Institute for Diabetes, Zagreb, Croatia).

Steering committee members

J. H. Fuller (London), B. Karamanos, Chairman (Athens), A.-K. Sjolie (Odense), N. Chaturvedi (London), M. Toeller (Düsseldorf), G. Pozza, co-chairperson (Milan), B. Ferriss (Cork), M. Porta (Turin), R. Rottiers (Ghent) and G. Michel (Luxembourg).

Co-ordinating centre

J. H. Fuller, N. Chaturvedi, J. Holloway, D. Webb and L. Asbury, University College London, UK.

Central laboratories

G.-C. Viberti, R. Swaminathan, P. Lumb, A. Collins, S. Sankaralingham and M. A. Crook, Guy's and St Thomas' Hospital, London, UK.

Retinopathy grading centre

S. Aldington, T. Mortemore and H. Lipinski, Royal Postgraduate Medical School, Imperial College London, UK.

Nutrition co-ordinating centre

M. Toeller, W. A. Scherbaum and F. A. Gries, Heinrich Heine University, Diabetes Research Institute and Department of Endocrinology, Diabetology and Rheumatology, Düsseldorf, Germany.
